# Transition of care in inborn errors of immunity

**DOI:** 10.1097/ACI.0000000000000948

**Published:** 2023-10-05

**Authors:** Susan Tadros, Siobhan O. Burns

**Affiliations:** a Department of Immunology, Royal Free London NHS Foundation Trust; b University College London Institute of Immunity and Transplantation, London, UK

**Keywords:** inborn errors of immunity, primary immunodeficiency, transition

## Abstract

**Purpose of review:**

This review outlines the principles of transition, summarizes current information about transition practices in inborn errors of immunity (IEI) and highlights general and specific considerations for transition of patients with these conditions.

**Recent findings:**

Recent surveys demonstrate the variability in access to and transition practices in IEI. Key challenges of transition in IEI from the perspective of healthcare professionals include lack of adult subspecialists, lack of access to holistic care and fragmentation of adult services. Limited research focused on IEI patient and carer perspectives highlight information gaps, poor coordination and difficulty adapting to adult healthcare structures as important challenges for smooth transition.

**Summary:**

Local policies and practices for transition in IEI are highly variable with limited assessment of outcomes or patient experience. There is a need for IEI-focused transition research and for development of national and international consensus statements to guide improved transition in IEI.

## INTRODUCTION

Inborn errors of immunity (IEI) are a group of heterogenous rare inherited disorders that include conditions with increased susceptibility to infection but can also be associated with complications including autoimmunity, inflammation and increased susceptibility to malignancy [1]. Primary immunodeficiencies (PID) make up the largest subset of IEI, which also encompasses autoinflammatory disorders (AID), diseases of immune dysregulation and monogenic forms of autoimmunity and bone marrow failure [2]. Many IEIs develop early in life and the combination of earlier diagnosis through newborn screening, improved monitoring and treatment of complications and expanded therapeutic options, including haematopoietic stem cell transplantation (HSCT), have led to increased life expectancy. As a result, higher numbers of adolescents and young adults with IEIs require transition of care from paediatric to adult services [3]. Although transition guidelines exist for a number of immunologically-mediated chronic conditions such as juvenile-onset rheumatic diseases and asthma, there is limited IEI focussed research and guidance [4,5].

This review outlines the principles of transition and specific challenges for transition of patients with IEI. It summarizes our current information about transition processes for IEI and highlights general and specific considerations for transition of these patients. 

**Box 1:**
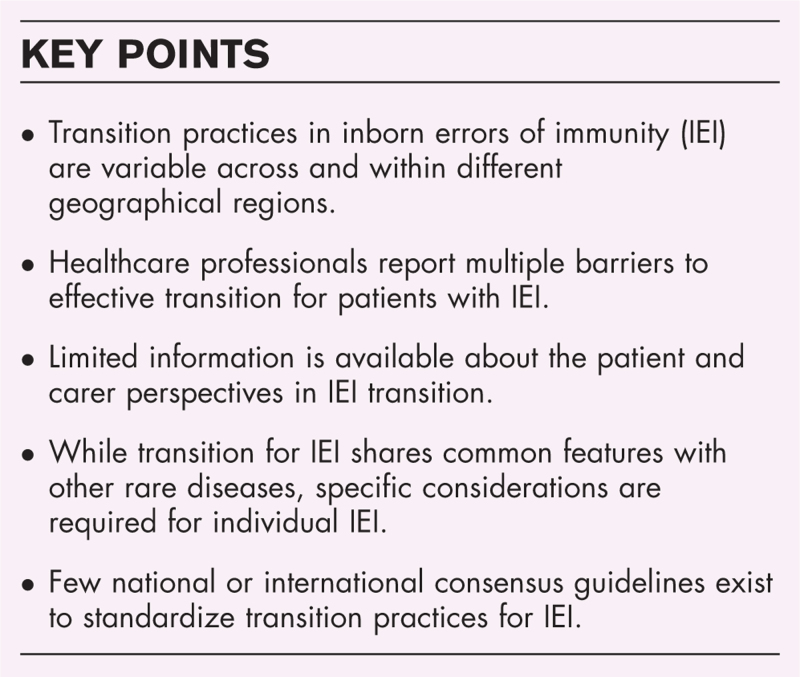
no caption available

## WHAT IS TRANSITION AND WHAT IS IT TRYING TO ACHIEVE?

Transition is defined by the Society for Adolescent Medicine as ‘the purposeful, planned process that addresses the medical, psychosocial and educational/vocational needs of adolescents and young adults with chronic physical and medical conditions as they move from child-centred to adult-oriented health-care systems’ [6]. The term and process of transition is distinct from ‘transfer’ or the physical move of care from paediatric to adult services. The transfer of care is an important aspect but transition of care describes a multifaceted, continuous process that aims to support the young person in gaining knowledge, skills and confidence to independently manage and become responsible for their own health [7^▪^,^8^].

**FIGURE 1:**
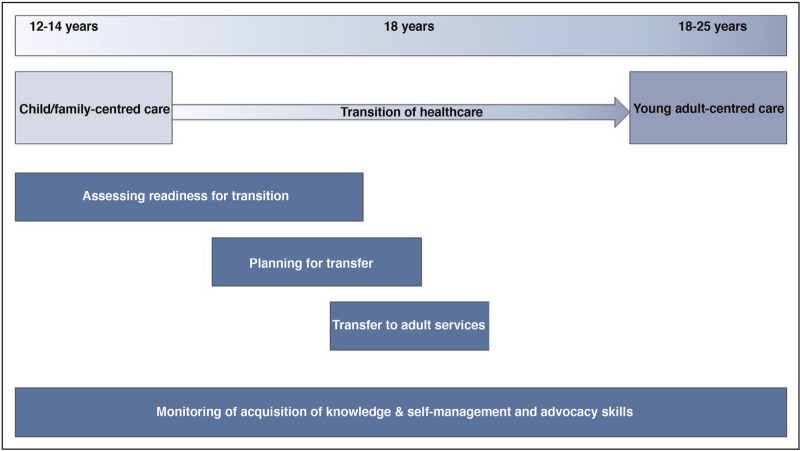
Key stages and components of transition.

Structured and standardized approaches to transitional care have been used. These include the ‘Ready Steady Go’ transition programme used in some National Health Services in the UK and the six core elements of the Got transition programme [9,10]. These programs are beneficial to set expectations for patients, families, and carers, and to provide guidance for clinicians as they enable adolescents and young adults to take ownership for their own care. Standardized approaches generally start around the age of 12 and outline several stages of transition that would be expected to occur sequentially until eventual transfer of care to adult services (see Fig. 1). The general principles of structured approaches include assessing readiness for transition, regular monitoring of knowledge and self-management skills, preparation and planning for transfer of care followed by the actual transfer to adult services. The process should be clearly laid out in a local policy or standard operating procedure and ideally electronic medical record systems should assist in the monitoring of knowledge and skills at each appointment and interaction with the young person [4,5,9,10,11^▪^]. Assessment of readiness for transition may include tools such as the transition readiness assessment questionnaire (TRAQ) [12,13]. Elements of transition should be mapped to the adolescent's clinical and developmental stage rather than to age alone. Transition skills checklists can be used to monitor progress with acquisition of knowledge and self-management skills [9].

When transition occurs well and is coordinated in an effective and meaningful way, this is associated with improved outcomes for young adults with chronic disease.

Poorer health outcomes are reported with unsuccessful transition or absence of a formal transition process. This has been demonstrated in other disease groups including HIV, systemic lupus erythematosus (SLE), inflammatory bowel disease and transplantation [14–18]. Examples of poorer health outcomes include moderate disease activity and gaps in care in patients with SLE, high graft failure rates in renal transplant recipients and increased requirement for surgery, poorer growth and increased flares in patients with inflammatory bowel disease [15–18].

## CURRENT STATE OF TRANSITION FOR INBORN ERRORS OF IMMUNITY

The importance of developing transition pathways and integration of transitional care in IEI is highlighted by patient organizations [19]. A key challenge for transition of IEI is the highly variable availability of specialist paediatric, and in particular, adult clinicians with appropriate disease knowledge across different regions. This is impacted by multiple factors including lack of medical training programs for physicians looking after patients with IEI and the organization and funding priorities of national organization of health services. The issue was highlighted in the PID Life Index survey of national patient organizations conducted by The International Patient Organization for Primary Immunodeficiencies (IPOPI) where PID transition care from paediatric to adult services was not available at all in 22/54 countries surveyed and only in specialized centres in 27/57 other countries [20]. In fact, only 3 countries reported that transition care for PID was available in most centres.

Recently, several surveys of healthcare professionals, have assessed the current state of transition practices underlining the variation in transition practice for IEI across different global regions. The European Reference Network on Rare Immunodeficiency, Autoinflammatory and Autoimmune Disease (ERN-RITA) Transition Working Group consortium surveyed paediatric centres in Europe caring for children with Primary Immunodeficiency (PID) and Autoinflammatory Disease (AID) and reported that the majority of centres had transition processes in place, provided at least one joint in-person or virtual appointment with the adult team prior to transfer and typically transitioned children with PID or AID to specialist adult PID or AID centres (86% and 77% respectively) [7^▪^]. This likely reflects the fact that the majority of responding paediatric centres were relatively large (caring for >100 patients with PID) with access to specialist adult centres, which may not be generalizable across Europe. In contrast, a South East Asia Primary Immunodeficiencies (SEAPID) consortium survey of paediatric centres caring for patients with PID in 7 countries across South East Asia found that 3/7 countries reported no practice of transition care with paediatricians continuing to be the primary provider of care for in some countries [21^▪▪^]. In North America, a survey of allergists and immunologists among members of the American Academy of Allergy Asthma and Immunology (AAAAI) and Clinical Immunology Society (CIS), highlighted that over half of clinicians caring for patients with PID transitioned their own paediatric patients and retained care of their adult PID patients, reflecting that the majority of respondents combined had subspecialty training encompassing both paediatric and adult Allergy and Immunology [22^▪▪^].

Despite variability in access to and practice of transition seen in these three surveys, similarities emerged regarding current difficulties in transition practice for IEI across all regions. Common themes included fragmentation of adult services, lack of adult subspecialists, reluctance of patients and their care givers to transfer from their paediatrician, poor engagement with health professionals, lack of holistic care at both paediatric and adult centres to support patients and their families and lack of time and resource to prepare transition documentation [7^▪^,^21^^▪▪^,^22^^▪▪^]. Formal local policies were variably in place and lack of national/international consensus statements or guidelines for transition of primary immunodeficiency patients was cited by all surveys as a gap.

## THE PATIENT AND CAREGIVER PERSPECTIVE

Our current assessment of transition care for IEI lacks information about the patient and family or carer perspective. A very recent Canadian study reported semi-structured interviews in a small group of young people looked after in an adult immunology clinic (most of whom had a diagnosed IEI; mean age 21 years), along with care givers and healthcare professionals [23^▪▪^]. In general, patients and carers report common challenges experienced during transition [24,25]. These include perceived differences in quality of care, knowledge and experience of adult healthcare providers, anxiety about leaving trusted relationships established with their paediatric team, as well as the change in environment and structure of adult healthcare services [23^▪▪^,^24^,^25^]. Uncoordinated transitions, with poor handover of information, emerged as a major theme that generated stress for patients and caregivers in addition to difficulties in breaking ties with the paediatric team and adjusting to differences in attitudes and approaches in adult healthcare systems. Many of the participants did not feel that they had received sufficient information prior to transfer and experienced unpleasant and unexpected changes when they move to adult services. Negative experience was compounded by the fact that transition typically coincides with an important developmental stage for youth as they move into adulthood and assume increasing independence [23^▪▪^].

Key recommendations from the study were that there should be a joint meeting with both paediatric and adult teams prior to transfer, that young people should meet healthcare professionals prior to transfer and should be provided with documentation about differences between both systems. In addition, clear instructions should be given to patients and their caregivers about where to go in case of medical emergencies, should have a transition coordinator and a dedicated nurse as fast as possible. Connecting patients and carers with a network of youth and caregivers going through transition or recently transferred was also recommended [23^▪▪^].

A separate study at the United States National Institute of Allergy and Infectious Diseases captured the transition experience of 33 young adult patients with chronic granulomatous disease (CGD) as they transitioned from paediatric to adult services within the Institute [26]. In contrast with the Canadian study, the majority of patients perceived their transition as uneventful, which may reflect differences in the study populations or the advantage of co-location of paediatric and adult services. Despite this, some patients reported not feeling included in the planning process and not feeing fully informed about the changes to expect when moving to adult care.

## TOWARDS IMPROVED TRANSITION FOR INBORN ERRORS OF IMMUNITY

Like other rare diseases, IEI presents additional challenges for transition that are not frequently encountered with common disorders, such as poor awareness amongst healthcare professionals and paucity of licenced drug therapies. In addition, IEI are heterogenous conditions that frequently manifest with complex multisystem disease complications and/or learning difficulties which require multidisciplinary approaches for successful management. Furthermore, a significant proportion of children with severe IEI have undergone hematopoietic stem cell transplant during childhood and a small but growing number have had autologous gene therapy or thymus transplants (mainly through clinical trials) with variable long-term treatment effects and/or ongoing disease manifestations. Typically, patients require transition to multiple adult specialties which may not be located in the same hospital and usually do not occur contemporaneously, confounding smooth communication and introducing clinical risk during the transfer period.

General principles and considerations for transition of patients with IEI are summarized in Table 1.

**Table 1:** General principles and considerations for transition of patients with IEI

Transition should start as early as possible and be tailored to the adolescent/young adult as an individual. It should be developmentally appropriate rather than age-specific and be patient-focused [27]
Information about what transition involves and aims to achieve should be provided to adolescents/young adults and their carers
Provision of and easy access to high quality, up-to-date, IEI specific information to promote understanding of a young person's condition including disease-manifestations and complications, treatments and their side effects. Where appropriate, information about inheritance of disease and impact of condition and treatments on fertility should also be provided.
Highly coordinated, multidisciplinary and multispeciality transition is required for complex, multisystem disease, ideally facilitated by a transition coordinator [23^▪▪^,^28^]
Transition should utilise a holistic approach with access to psychological and social support [29]
Transition should include clear written and verbal handover of patients from paediatric to adult team with inclusion of key information [29]. Ideally at least one joint consultation with the patient, paediatric and adult teams should occur to facilitate this process and provide the young person with an opportunity to meet the adult team prior to transfer [30]
Transition partnerships should be established to ensure coordinated and safe transition with ongoing communication between paediatric and adult teams after transfer of young adult. Ideally patients should be transitioned to a young adult clinic [3,7^▪^,^19^]
Diagnostic revaluation of those without a clear diagnosis should be conducted prior to transfer of care [29]
Arrangements should be made for long term follow-up of young adults in clinical trials

IEI, inborn errors of immunity.

In addition, there are specific considerations that need to be taken into account for transition of specific IEI. These were highlighted in the recent Italian PID network consensus guidelines and relate to the known complications of individual conditions [29]. For example, the risk of later neuropsychiatric disorders in DiGeorge syndrome requires additional vigilance as it may present in the first instance to the responsible immunologist [31,32]. Similarly, malignancy risk for patients with DNA-repair defects is higher than for other forms of IEI and may include nonhaematological as well as haematological cancers (such as breast cancer in Ataxia Telangiectasia) which require monitoring not typically included in IEI long-term follow up [33]. Some specific transition considerations are summarized in Table 2.

**Table 2:** Transition considerations for specific IEI

Specific transition considerations	Examples of IEIs where applicable
Learning disabilities assessment and support	22q deletion syndrome, adenosine deaminase (ADA) SCID
Neuropsychiatric vigilance, assessment and follow-up	22q deletion syndrome
Consideration of HSCT and regular review of indications for HSCT in adolescence/early adulthood	Various IEIs e.g. CGD, combined immune deficiency acquiring complications

CGD, chronic granulomatous disease; HSCT, haematopoietic stem cell transplantation; IEI, inborn errors of immunity.

## CONCLUSION

Recent surveys provide insight into and highlight the variability in access to and transition practices in IEI. Many of the considerations and some of the challenges in transition in IEI are shared with other rare diseases whilst others are relevant to specific IEIs. Recent publications in this area highlight some of the patient and carer perspectives and start to generate consensus on what transition for IEI should include. Further IEI focused research and guidance is required to ensure transition practices are optimized and patient-focused.

## Acknowledgements

*None.*

### Financial support and sponsorship

*None.*

### Conflicts of interest

*There are no conflicts of interest.*

## REFERENCES AND RECOMMENDED READING

Papers of particular interest, published within the annual period of review, have been highlighted as:

▪ of special interest
▪▪ of outstanding interest
